# Predicting financial distress in TSX-listed firms using machine learning algorithms

**DOI:** 10.3389/frai.2024.1466321

**Published:** 2024-11-27

**Authors:** Mark Eshwar Lokanan, Sana Ramzan

**Affiliations:** ^1^Faculty of Management, Royal Roads University, Victoria, BC, Canada; ^2^University Canada West, Victoria, BC, Canada

**Keywords:** machine learning, artificial intelligence, financial distress, M-score model, earnings management

## Abstract

**Introduction:**

This study investigates the application of machine learning (ML) algorithms, a subset of artificial intelligence (AI), to predict financial distress in companies. Given the critical need for reliable financial health indicators, this research evaluates the predictive capabilities of various ML techniques on firm-level financial data.

**Methods:**

The dataset comprises financial ratios and firm-specific variables from 464 firms listed on the TSX. Multiple ML models were tested, including decision trees, random forests, support vector machines (SVM), and artificial neural networks (ANN). Recursive feature elimination with cross-validation (RFECV) and bootstrapped CART were also employed to enhance model stability and feature selection.

**Results:**

The findings highlight key predictors of financial distress, such as revenue growth, dividend growth, cash-to-current liabilities, and gross profit margins. Among the models tested, the ANN classifier achieved the highest accuracy at 98%, outperforming other algorithms.

**Discussion:**

The results suggest that ANN provides a robust and reliable method for financial distress prediction. The use of RFECV and bootstrapped CART contributes to the model’s stability, underscoring the potential of ML tools in financial health monitoring. These insights carry valuable implications for auditors, regulators, and company management in enhancing practices around financial oversight and fraud detection.

## 1 Introduction

Financial statement fraud (FSF) is a significant issue in corporate governance, often linked to instances of financial distress. Notable examples include Canadian companies like Livent and Nortel Networks, as well as international firms such as Enron, WorldCom, and Parmalat, where financial distress led to manipulative practices that violated generally accepted accounting principles (GAAP) and International Financial Reporting Standards. Although earnings management and fraud are distinct concepts, the literature demonstrates a strong correlation between financial distress and the likelihood of fraudulent financial reporting (Perols, 2011; Ramírez-Orellana et al., 2017; Trompeter et al., 2013).

The advent of machine learning (ML) offers promising opportunities to address these issues by enabling auditors and regulators to proactively identify high-risk firms and prevent financial fraud. Specifically, supervised learning within ML, including algorithms like logistic regression, support vector machines (SVM), and artificial neural networks (ANN), has shown potential in predicting financial distress and detecting earnings manipulation. This purpose of this research is to explore how these ML algorithms, alongside classification and regression trees (CART) with bootstrapping techniques, can be utilized to predict financial distress and identify instances of earnings manipulation using the Beneish M-score as a key indicator.

We aim to answer the central research question: “How can ML algorithms be leveraged to predict financial distress and detect earnings manipulation in companies using the Beneish M-score?” We employ these advanced techniques to forecast financial distress among companies listed on the Toronto Stock Exchange (TSX). Our central focus revolves around developing a model that not only predicts financial distress but also sheds light on potential earnings manipulations, leveraging readily available financial statement data from TSX-listed companies. We recognize the inherent connection between financial distress and earnings manipulation, as companies facing financial difficulties are more likely to engage in such practices (Ding et al., 2023; Fang et al., 2017; Farooq and Qamar, 2019). Therefore, our research encompasses both aspects to provide a comprehensive understanding of these intertwined phenomena.

This research contributes to the growing field of financial distress prediction by adopting a multi-faceted approach, integrating four distinct methodologies: predictive modeling, recursive feature elimination with cross-validation (RFECV), an AI feedforward model (specifically an ANN), and CART with bootstrapping.

In this paper, “feedforward model” refers to the architecture of the neural network, where information flows in one direction—from the input layer through the hidden layers to the output layer. This structure is commonly used for predictive tasks that do not involve temporal sequences or dependencies. By using a feedforward approach, we ensure that the model processes data in a straightforward manner, without any cyclical or recurrent connections between layers.

While each technique is established, our study’s innovation lies in combining these methods within a single framework to achieve a more comprehensive and robust prediction of financial distress. Specifically, the application of CART with bootstrapping is relatively new in the financial distress literature, providing a fresh perspective that enhances model stability and accuracy. This approach also addresses common issues like overfitting by improving generalization across different datasets.

By expanding beyond traditional predictive modeling and the commonly used AI techniques in financial distress research, this study fills a gap identified by previous researchers. Our work demonstrates the effectiveness of advanced ML techniques in diverse contexts, particularly using data from the TSX, thereby broadening the applicability of these methods beyond the Asian markets that have been the focus of much prior research.

Moreover, our study introduces RFECV and CART with Bootstrapping as innovative approaches in this field, offering alternatives to the conventional predictive modeling algorithms. We empirically assess these methods, highlighting the importance of considering the unique characteristics of specific datasets and problem domains when developing predictive models. This emphasis on context-specific solutions rather than generic predictive modeling insights underscores the value of our approach in addressing the complexities of financial distress prediction.

The remainder of this paper is organized as follows: Section 2 reviews the existing literature, emphasizing contemporary audit methodologies and the use of ML in research on predicting financial distress. Section 3 details the experimental design, covering aspects such as data collection, preprocessing, the algorithms used, and performance metrics. Section 4 presents the results and offers a thorough analysis of the findings. Lastly, Section 5 concludes the paper and highlights potential directions for future research.

## 2 Literature review

There has been a lot of work on financial distress prediction, with various methods and models developed to assess the likelihood of a company experiencing financial distress. Some of this scholarship has focused on bankruptcy prediction (du Jardin, 2015; Fedorova et al., 2022; Mai et al., 2019), while others focused on financial distress (Campa and Camacho-Miñano, 2015; Hammami and Hendijani Zadeh, 2022; Veganzones et al., 2023; Zhou et al., 2022). In the early stages of research, financial distress prediction models were primarily based on ratios of financial quantities. This approach was pioneered by researchers such as Altman and Beneish, who developed models like the Altman Z-Score and M-Score, respectively (Altman et al., 2017; Beneish, 1999). Later on, machine and deep learning techniques were also incorporated to enhance the accuracy of financial distress prediction models (Chen and Du, 2009; Song et al., 2023; Tang et al., 2020; Zhao et al., 2023). These models have been widely used in various domains, such as assessing credit risks for financial institutions, aiding investors in making informed decisions, supporting regulatory authorities in monitoring financial stability, and helping managers in evaluating corporate financial health, including the prediction of financial distress.

The literature on financial distress prediction is well-established and continues to evolve, with ongoing research and development focusing on improving the accuracy and effectiveness of these prediction models. In this literature review, we shift gears and discuss the challenges of detecting financial statement manipulation and the crucial role of auditors in maintaining financial credibility and detecting irregularities. We highlight the prevalence of fraud and the need for stakeholders to access accurate financial information through independent audits. The limitations of traditional auditing procedures and the potential of ML based technologies to identify financial distress predictors are also emphasized.

### 2.1 Current audit methodology and distress prediction

Financial statement manipulation is becoming increasingly prevalent due to auditors’ inability to identify red flags of fraud. Typically, fraudsters exaggerate positive financial positions and conceal negative ones, making it difficult to detect manipulation in financial statements (Blay et al., 2007; Firth et al., 2011; Hartwig et al., 2017; Hilal et al., 2022; Lokanan and Sharma, 2022). Companies manipulate their earnings either by inflating their revenues or deflating their expenses (Eulerich et al., 2018; Filip and Raffournier, 2014; Perols and Lougee, 2011). This manipulation not only limits stakeholders’ access to accurate financial information during corporate distress (DeZoort and Harrison, 2018; Khaksar et al., 2022) but also poses severe consequences for investors and creditors. To address these challenges and ensure the availability of reliable financial data, independent audits have become paramount (DeZoort and Harrison, 2018; Eulerich et al., 2018; Khaksar et al., 2022).

Auditors play a pivotal role in upholding the integrity of financial statements and must continually refine their audit procedures to effectively detect irregularities and manipulations (Akther and Xu, 2020; Oyerogba, 2021). However, they often face challenges in identifying transaction omissions due to inadequately designed audit procedures (Hamilton and Smith, 2021). Given this context, both financial statement users and auditors should prioritize vigilant monitoring of companies to uncover discrepancies, anomalies, or overstated positives in their engagements (Lokanan and Sharma, 2022). Rigorous monitoring can serve as early warning signs, enabling timely actions to mitigate the adverse effects of distorted financial statements (Aslam et al., 2022; Aviantara, 2021; Dechow et al., 1996).

While the engagement approach remains the standard practice in auditing financial statements, there is growing recognition of its limitations in identifying red flags that may indicate potential fraud (Aubert et al., 2019; DeZoort and Harrison, 2018; Sun et al., 2014). Traditional measures like the fraud triangle, as outlined in SAS No. 99, evaluate pressure, opportunity, and rationalization to detect fraudulent activities (Aubert et al., 2019; Davis and Pesch, 2013; DeZoort and Harrison, 2018). However, these methods have been criticized for their limited ability to assess patterns of financial distress that create the conditions for fraudulent activities (Davis and Pesch, 2013; Firth et al., 2011; Lokanan, 2018; Nasir et al., 2019).

As a result, researchers have concluded that more comprehensive investigative strategies are necessary to identify suspicious activities and prevent future instances of fraud (Lokanan, 2015; Morales et al., 2014). One promising approach involves the incorporation of ML technologies to identify predictors of financial distress (Campa and Camacho-Miñano, 2015; Mselmi et al., 2017; Zhao et al., 2023). ML-based approaches should be integrated into the audit process to provide auditors with additional insights and indicators that can help them focus their efforts more effectively. These advanced techniques, while not a replacement for traditional audit practices, offer valuable supplementary tools to enhance financial analysis and auditing, enabling a more effective response to the challenges posed by financial manipulation and distress detection.

### 2.2 Machine learning for financial distress prediction

Although the application of computational technology is in its early stages, recent studies have shown that ML hold a significant advantage over traditional audit approaches in detecting red flags of fraud in financial statements. More specifically, these researchers found that ML-based algorithms yield high precision, sensitivity, and accuracy scores in detecting fraud (Achakzai and Peng, 2023; Bao et al., 2020; Zhao et al., 2023). Essentially, ML-based tools are more adept at detecting anomalies and red flags of fraud in financial statements than the traditional sampling approach (Hilal et al., 2022; Mselmi et al., 2017). More specifically, researchers have found that ML can identify and report more accurately on true positives and negatives, lending greater support to the use of analytics in fraud risk management processes (Blay et al., 2007; Kuizinienė et al., 2022; Lokanan and Sharma, 2022; Qiu et al., 2021).

Researchers have employed various ML techniques to predict fraud in financial statements. Techniques such as neural networks, SVM, and decision trees have been employed to predict anomalies and uncover fraudulent accounting practices in organizations (Achakzai and Peng, 2023; Hilal et al., 2022; Kim and Kogan, 2014; Mselmi et al., 2017). The application of ML- technologies enables more effective processing of larger volumes of data, while generating more accurate predictions to reduce instances of false positives (Achakzai and Juan, 2022; Hilal et al., 2022; Kim and Kogan, 2014). Others have found that ML models capture patterns and trends in the data and provide opportunities to gain deeper insight into potential warning signs of fraud within financial statements that may often be missed by human analysts (Cho et al., 2020; Hajek and Henriques, 2017).

Integrating ML-based analytics with traditional statistical methods has proven more efficient than manual analysis to detect anomalies in financial statements (Achakzai and Juan, 2022; Albizri et al., 2019; Alden et al., 2012; Kim and Kogan, 2014). In particular, research has found that combining ML with the existing Beneish M-score produces superior results compared to manual computation as a means of predicting financial distress (Papík and Papíková, 2022). Subsequent work by Papík and Papíková (2022) also showed that predictive models developed using data mining and ML effectively identify accounting fraud at a much higher rate than traditional methods. By combining and utilizing the best features of ANN, SVM, and ensemble models, these algorithms have proven to be highly effective in predicting financial distress and detecting fraud (Mselmi et al., 2017; Nasir et al., 2021; Zhao et al., 2023). This paper adds to this stream of literature by presenting an investigation in which ML technology are used to predict financial distress and detect earnings manipulations using data from the TSX. The Beneish M-score serves as a proxy for manipulation. Based on the preceding literature review, we attempt to predict financial distress and detect earnings manipulations by asserting that:


$$\text{Financial}\;\text{Distress}=f\;\left(\begin{array}{l} \text{profitability},\;\text{liquidity},\;\text{efficiency},\;\text{solvency},\\ \text{and}\;\text{operational}\;\text{performance}\;\text{indicators} \end{array}\right)$$

Where,

“*f*” stands for “function” and indicates that financial distress is a function of, or is determined by, the variables listed—profitability, liquidity, efficiency, solvency, and operational performance indicators.

## 3 Experimental setting

This paper employs classification models to assess financial distress and detect earnings manipulation in TSX-listed companies. Our analysis aims to reveal critical factors for predicting financial distress and assess the likelihood of future distress occurrences. Our objective is to identify the most relevant features for predicting financial distress and build models that accurately classify firms at risk based on various financial ratios. This predictive approach has demonstrated effectiveness in the early detection of financial distress and warning of earnings manipulation (Mselmi et al., 2017; Sun et al., 2014; Zhao et al., 2023).

### 3.1 Data collection

The dataset used in this study was sourced from the TSX, one of the world’s largest stock exchanges and a constituent of the S&P/TSX Composite Index, encompassing the top 500 Canadian companies. This exchange boasts a substantial market capitalization of CAD$2.1 trillion and is home to numerous prominent Canadian banks, insurance companies, and financial institutions. The Toronto Stock Exchange (TSX) houses Canada’s leading publicly listed corporations, including diverse industries like banking, insurance, and financial services. A sample size of 464 enterprises was established by using meticulous data screening to exclude companies with missing data. These companies were selected based on their substantial contribution to the Canadian economy and their public accessibility of financial information. These enterprises function under a legal and political framework specific to Canada and may vary from the social, cultural, and political atmospheres experienced by companies in other areas. This differentiation enables the research to provide valuable contributions to financial distress prediction in the Canadian market. However, the conclusions of this study may have limited relevance to other international markets.

Compiled from the fiscal year-end financial reports as of March 31, 2021, the dataset covers a time significantly affected by the COVID-19 epidemic. The significance of this context cannot be overstated, since the epidemic has potentially obscured the distinction between financially distressed and financially stable companies, therefore confounding the investigation. The choice of the sample size was determined by the availability of comprehensive financial data for these firms, therefore assuring the reliability of the model’s predicted outcomes. The dataset comprises essential financial measures that are crucial for forecasting financial difficulties, including profitability, liquidity, solvency, and efficiency metrics. Hence, the aim of this study is to investigate the efficacy of machine learning algorithms in accurately forecasting financial hardship, with a specific emphasis on the distinctive setting of the TSX.

### 3.2 Variables and measurements

#### 3.2.1 Independent variables

The independent variables used to predict financial distress, as detailed in Supplementary Appendix 1, encompass a range of essential indicators of corporate financial health, including profitability, liquidity, efficiency, solvency, and operating performance. Given their well-established status as standard metrics for assessing a company’s performance, we refrain from delving extensively into these common practices. Instead, our focus lies in elucidating their applicability for ML models in the context of financial distress prediction. These ratios serve as vital input features for our predictive models, enabling us to harness the power of ML to identify patterns, interactions, and predictive signals within these financial indicators.

#### 3.2.2 Profitability ratios

Profitability ratios, such as return on assets (ROA), gross profit margin (GPM), net profit margin (NPM), return on equity (ROE), and net profit to total assets (NPTA), have been recognized as potent predictors of financial distress (Demetriades and Owusu-Agyei, 2022; Fang et al., 2017; Hajek and Henriques, 2017; Sun et al., 2014). In the context of ML, these ratios play a pivotal role as they capture a company’s financial performance, offering valuable insights into its ability to generate revenues efficiently. These ratios act as data-driven inputs, enabling ML models to discern intricate relationships between profitability metrics and the likelihood of financial distress. Profitability ratios are one of the most fraud-sensitive types of ratios in that their values differ significantly between financially distressed companies (true positives) and non-distressed companies (false positives), making them useful for predicting financial distress.

#### 3.2.3 Liquidity ratios

Liquidity ratios evaluate a firm’s ability to meet short-term financial obligations (Hajek and Henriques, 2017; Huang et al., 2017). Some liquidity ratios used in previous research to predict financial distress are the current ratio (CR), quick ratio (QR), days receivable turnover (AccTR), working capital to total assets (WCTA), cash-to-current liabilities (CCL), and cash-to-total assets (CTA) (Albizri et al., 2019; Fang et al., 2017; Hajek and Henriques, 2017; Song et al., 2014). In the realm of ML, these ratios are invaluable as they provide a real-time snapshot of a company’s ability to meet its immediate financial obligations. Incorporating these liquidity ratios into our predictive models allow us to leverage ML to uncover trends and identify liquidity-driven indicators of financial distress.

#### 3.2.4 Efficiency ratios

Previous research has shown that efficiency ratios, such as accounts days receivable turnover (AR), days accounts payable turnover (AP), asset turnover (AT), and inventory turnover (InvT), have been widely recognized as valuable indicators of a company’s financial health (Albizri et al., 2019; Dimitropoulos and Asteriou, 2009; Fang et al., 2017; Habib et al., 2020; Zainudin and Hashim, 2016). In the context of ML, these ratios are of paramount importance as they offer insights into the competitive landscape and profitability of a firm. Their incorporation in our analysis enables us to harness ML to uncover patterns related to operational efficiency and their implications for financial distress. By doing so, we enhance our predictive models’ ability to identify nuanced indicators of distress rooted in operational metrics.

#### 3.2.5 Solvency ratios

Solvency ratios, such as debt to equity (DEQ), total liabilities to total assets (TLTA), net profit to total liabilities (NPTL), cash to total liabilities (CTL), current assets to total assets (CATA), and current liabilities to total assets (CLTA), have been used to assess organizations’ probability of default (Albizri et al., 2019; Craja et al., 2020; Fang et al., 2017; Huang et al., 2017). Within the fields of ML, these ratios provide valuable insights into a company’s ability to withstand financial challenges. By incorporating solvency ratios into our analysis, the ML models can be leveraged to explore the complex relationships between financial structure and financial distress. This approach enhances the capacity of our models to identify subtle yet crucial indicators of potential financial distress in companies.

#### 3.2.6 Operating performance

Others have employed earnings and cash flow ratios to assess the operational performance of companies in the context of financial distress prediction. Ratios such as Earnings Before Interest and Taxes (EBIT), Earnings Before Interest, Taxes, Depreciation, Amortization, and Rent (EBITDAR), and Earnings Before Interest, Taxes, Depreciation, and Amortization (EBITDA) have been widely used in financial distress research (Albizri et al., 2019; Campa and Camacho-Miñano, 2015; Li, 2016). In the context of ML, these ratios provide crucial inputs that allow us to assess the financial health of a company based on its operational efficiency. By incorporating these ratios into our analysis, the ML models are able to delve deeper into the operational dynamics that underpin financial distress prediction.

#### 3.2.7 Control variables

Several control variables were used to isolate and measure the specific effect of the independent variable on financial distress while keeping other relevant factors constant (Bernerth and Aguinis, 2016; Farber, 2005). In this study, a comprehensive set of control variables was included to evaluate the precise impact of profitability, liquidity, efficiency, solvency, and operational variables on predicting financial distress. These variables encompassed the firm’s credit rating, R-Score, Enterprise Value (EV), Market Capitalization (MarketCap), revenue growth (Rev_G), net income growth rates (NI_G), dividend growth (D_G), Corporate Governance Score (CG_Score), Audit and Risk Oversight (ARO), Board Structure (BoardS), Shareholder Relations (ShareR), and Compensation (Comp). By including these control variables in our analysis, we aimed to account for potential confounding factors and variations in firm characteristics (Becker et al., 2016; Bernerth and Aguinis, 2016). The inclusion of control variables provides insights into how the chosen financial and operational indicators worked alone and together to predict financial distress.

### 3.3 Dependent variable

The dependent variable for this project is the Beneish M-score (Beneish, 1999), which is used as a proxy for financial distress, given that companies often manipulate earnings when they are under financial pressure. The Beneish M-score is one of the best accounting indicators for predicting financial distress and detecting earnings manipulation (Albizri et al., 2019; Aviantara, 2021; Maniatis, 2022; Repousis, 2016). The Beneish M-score is a probabilistic model that uses financial ratios, which are categorized into eight variables, to detect earnings manipulation in a company’s earnings. Companies with higher M-Scores tend to manipulate their earnings because they are experiencing financial distress. Others noted that companies could use the “M-score models and data mining for an early indication of financial distress or red flags for detecting financial fraud” (Tarjo and Herawati, 2015, p. 2). In a more recent study of the M-score model, Beneish and Vorst (2022) claimed that a cost-based assessment of models is preferable to traditional model comparison measures. We use the M-score as an indicator of potential manipulation in financial statements. In our analysis, a higher absolute value of the M-score, whether positive or negative, is associated with a greater likelihood of manipulation. To operationalize this, we set a dummy variable to 1 for manipulators when the M-score is smaller than −2.22 and to 0 for non-manipulators when the M-score is greater than −2.22. Therefore, our approach considers both positive and negative M-score values as potential indicators of manipulation, with higher absolute M-score values suggesting a stronger likelihood of manipulation. The formula to represent financial distress in the earnings manipulation modeling is shown in Equation 1:


(1) $$y=\{\begin{array}{c} 1\;\;\text{Financial}\;\text{distress}\;\\ 0\;\text{No}-\text{financial}\;\text{distress} \end{array}$$

### 3.4 Machine learning workflow

**Figure 1:**
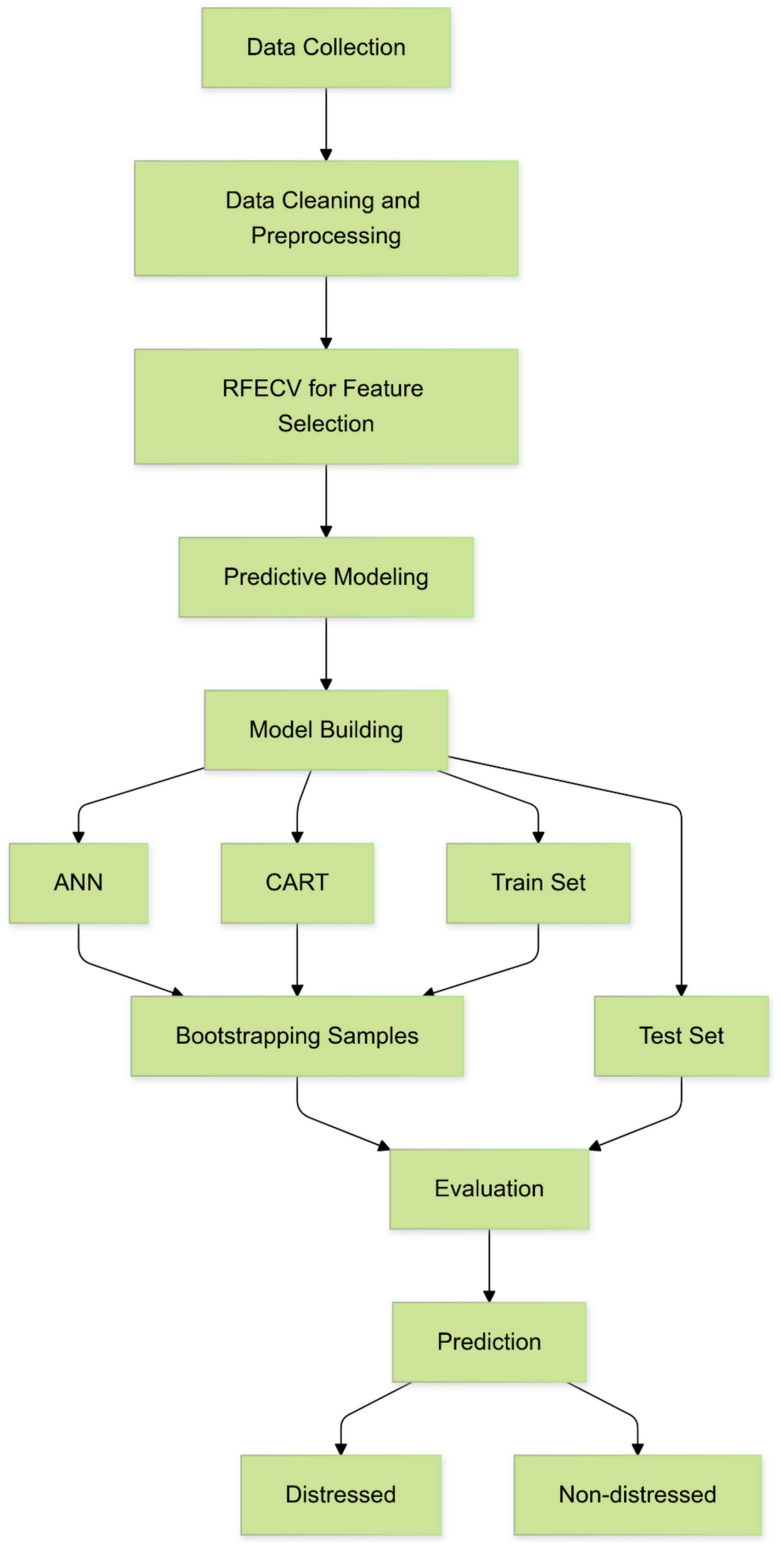
Overview of the machine learning workflow.

Figure 1 illustrates the workflow employed in this project to construct a classification model capable of assessing whether a firm has engaged in financial manipulation. The process begins by gathering data suitable for building the classification model, followed by preprocessing the features to identify outliers or noise in the data. Preprocessing also includes standardizing the data so that all observations are within the same range. Subsequently, the data is split into train and test sets, with the test set acting as an unseen sample from which to evaluate model performance. Finally, the evaluation of the model takes place using validation techniques on the test set before making the final prediction.

#### 3.4.1 Data cleaning and preprocessing

Data cleaning is a critical step in the data preprocessing pipeline and is essential for obtaining meaningful and reliable insights from the data. A check for duplicates indicates that there were none in the dataset. The feature “Rating” represents the ratings from Moody’s and ranges from “Aaa” to “C.” These letters were replaced with integers, where “Aaa” = 1 and “C” = 9 the lowest rating. Outliers were identified using the Z-score method, with a threshold of ±3 (Salgado et al., 2016). Data points exceeding this threshold were considered potential outliers (Chikodili et al., 2021) and removed to prevent them from disproportionately influencing the model’s performance and skewing results. While outliers can sometimes indicate distressed companies due to atypical or suspicious behavior, removing extreme outliers ensures that the model generalizes effectively across various scenarios. By focusing on the M-score as our primary measure of distress, we ensure that our model is identifying meaningful indicators of financial instability rather than being misled by extreme outliers that may obscure the true patterns of distress. Removing these outliers helps maintain the integrity of the dataset, allowing the model to focus on the relevant predictors of distress as defined by the M-score, without being disproportionately influenced by unrelated anomalies. Thus, while outliers may sometimes reflect distress, the M-score provides a more accurate and robust method for detecting such conditions.

Missing values were imputed using the K-nearest neighbors (K-NN) method, which is particularly effective when missing data is ≤40% (Zhang, 2012). Multicollinearity was addressed by removing any features with a Pearson correlation of 0.70 and above from the dataset. While certain preprocessing steps may imply normality, the robustness of our approach does not strictly depend on this assumption. We also performed a variance inflation factor analysis to eliminate features that were highly correlated within the dataset.

The dataset exhibited a notable imbalance, with approximately 88% of the firms categorized as “not distressed” and the remaining 12% classified as “distressed.” To rectify this imbalance and create a more equitable representation of both classes, we employed the SMOTE+ENN technique. SMOTE+ENN combines the Synthetic Minority Over-sampling Technique (SMOTE) with the Edited Nearest Neighbors (ENN) algorithm to effectively address the class imbalance issue (Puri and Kumar Gupta, 2022). SMOTE generates synthetic samples for the minority class by interpolating between existing instances, thus augmenting the distressed samples in the dataset (Lokanan, 2023). Subsequently, the ENN algorithm identifies and eliminates noisy or redundant data points to enhance the overall quality of the dataset (Sisodia et al., 2017). This process ensures that the models have a more balanced and representative dataset to learn from, ultimately improving their predictive performance in identifying financial distress. As seen in Equation 2, the financial distress class consists of only 12% of the labeled data. After oversampling, the train and test dataset had equal samples (195 instances) of class 1 and class 0.


$$\text{Financial}\;\text{distress}\;{\left(Fd\right)}_{\text{Yes}}=\frac{\left(\text{Total}\;\text{transactions}\right)}{\left(\text{Distress}\;\text{transactions}\right)}*100$$

(2) $$Fd_{\text{yes}}=\frac{56}{464}*100=12\%$$

The min-max scaling technique was applied to the dataset to normalize the features, ensuring that they all fall within the same range. Normalization is a crucial preprocessing step in ML as it compresses all the variables to a common scale, preventing certain features from dominating others during the modeling process (Islam, 2021; Singh and Singh, 2020). Min-max scaling specifically transforms the values of each feature to lie between a specified range, typically 0 and 1. By compressing the variables between 0 and 1, min-max normalization helps to maintain the integrity of the data’s distribution while ensuring that all attributes contribute equally to the modeling process.

#### 3.4.2 Types of experiments

As can be seen in Table 1, the experiments conducted in this study encompass a diverse set of classifiers, algorithms, and techniques. Linear classifiers, such as logistic regression, are employed for predictive modeling. Ensemble classifiers, notably random forest, serve a dual purpose, facilitating both predictive modeling and feature selection. Tree-based classifiers, including decision trees and CART, are utilized for predictive modeling and RFECV. Finally, neural networks, specifically ANN, are harnessed for predictive modeling. These experiments provide a comprehensive evaluation of the effectiveness of these various methods in predicting financial distress within the specific context of TSX-listed firms.

**Table 1:** Types of experiments.

Classifiers	Algorithms	Predictive	RFECV	Bootstrapping
Linear	Logistic regression	x	x	
Ensemble	Random forest	x	x	x
Tree-Based	Decision tree	x	x	
	CART	x	x	x
Neural networks	ANN	x		

To perform our experiments, we divided the dataset into two subsets: a training set comprising 75% of the data and a test, or holdout set containing the remaining 25%. The model was then trained using the training data, enabling it to make predictions for the response variable on the test data, ensuring evaluation based on unseen observations. By randomly selecting samples from the dataset, we prevent any inherent data ordering from biasing our results. We also employed GridSearchCV to optimize the predictive models (Botchey et al., 2020; Lokanan, 2022).

#### 3.4.3 Hypermeters used for tuning

Table 2 presents a detailed account of the parameters used to fine-tune the ML models within the study. The tuning process was important in optimizing the models to achieve the best possible performance. While some models, such as the predictive and AI feedforward models, benefited from the automated tuning provided by GridSearchCV, it is important to note that this approach came at the cost of computational resources due to its resource-intensive nature. In contrast, manual tuning was used for the RFECV and CART models. This hands-on calibration process allowed for a more tailored and efficient configuration of RFECV and CART models, resulting in noticeable improvements in predictive accuracy compared to default settings. By manually adjusting the hyperparameters, we gained valuable insights into how each parameter influenced the model’s predictions, particularly in the context of financial distress prediction. The ability to exert precise control and customization over model parameters proved invaluable, especially when dealing with intricate financial datasets or when specific constraints or requirements needed to be met for accurate distress prediction. This approach provided a deeper understanding of the interplay between hyperparameters and model performance, ultimately leading to enhanced predictive capabilities.

**Table 2:** Hyperparameter tuning.

Algorithms	Predictive modeling with GridSearchCV
Logistic regression	“C”: 100, “max_iter”: 100, “penalty”: “l2,” “solver”: “newton-cg”
Decision tree	max_depth = 12, max_features = “auto,” min_samples_leaf = 4, splitter = “best”
Random forest	“max_depth”: 4, “max_features”: “auto,” “min_samples_leaf”: 2, “n_estimators”: 10
SVM	“C”: 100, “degree”: 2, “gamma”: 1, “kernel”: “rbf”
Algorithms	RFECV
Logistic regression	solver = “lbfgs,” penalty = “l2,” C = 1.0, max_iter = 100, class_weight = “balanced,” random_state = 42
Decision Tree	max_depth = 3,min_samples_split = 2,criterion = “gini,” random_state = 42
Random Forest	n_estimators = 100, max_depth = 3, min_samples_split = 2, criterion = “gini,” random_state = 42
SVM	kernel = “sigmoid,” C = 1.0, random_state = 42
Algorithms	Bootstrapping of Samples
CART	n_estimators = 100, max_depth = 3, max_features = “sqrt,” random_state = 42
Algorithms	Artificial Neural Network
AI feedforward model	“batch size”: 35, “number of epochs”: 100, “dropout_rate”: 0.0, “learn_rate”: 0.001, “unit”: 5, “number of hidden layers”: 3, “activation”: relu, “optimizer”: Adam

## 4 Algorithms and parameters tuning

### 4.1 Logistic regression

In our analysis, logistic regression was employed as the benchmark algorithm to assess model performance. Logistic regression is a supervised learning technique used to estimate or predict the likelihood of a binary event occurring in linear data (Lokanan and Liu, 2021; Lokanan and Sharma, 2022; Papík and Papíková, 2022). To evaluate the model’s predictions, we compared them against a predefined threshold. This threshold, which determines the classification of instances into binary categories, was established based on best practices and domain expertise. It plays a crucial role in interpreting model output and making predictions. The selection of this threshold was made by considering the specific context and objectives of the analysis and ensuring that it aligns with the desired balance between sensitivity and specificity in the classification task. The mathematical formula of logistic regression is represented in Equation 3:


(3) $$y=\frac{e^{\left(b_{0}+b_{1}*x\right)}}{\left(1+e^{(b_{0}+b_{1*x)}}\right)}$$

where,

*y* is the predicted output,

*b0* is the bias or intercept term, and

*b1* is the coefficient for the single input value (x).

Each column in the input data contains a *b* coefficient derived from the training data (Lokanan and Sharma, 2022; Papík and Papíková, 2022). Logistic regression is one of the most common ML classification techniques owing to its ease of implementation, functionality, and effectiveness in categorizing new information (Nasir et al., 2021; Wang and Song, 2011). To evaluate if a company is experiencing financial distress, logistic regression fits the data into a logistic function and compares their values against a predetermined threshold (Abbas et al., 2020; Papík and Papíková, 2022; Wang and Song, 2011).

#### 4.1.1 Decision tree classifier

Decision trees are a type of ML algorithm used to help solve regression and classification problems. The algorithm works by creating a tree-like structure, with each branch representing a different decision (Botchey et al., 2020; Sahin et al., 2013). The tree is created by splitting the dataset into smaller and smaller subsets until each subset only contains one data point. Once the tree is created, it can be used to predict new data points. Decision trees are generally highly accurate and are often used in predictive modeling tasks (Sahin et al., 2013; Tian et al., 2020). However, a single decision tree is often insufficient to produce effective results. For more accurate prediction, random forest classifiers (RFC) have proven to be significantly effective in fraud classification tasks (Aslam et al., 2022; Nami and Shajari, 2018).

#### 4.1.2 Random forest

The random forest algorithm is an extension of the decision tree algorithm that builds multiple trees and combines their predictions (Aria et al., 2021). The random forest classifier (RFC) is useful because it addresses nonlinearity in the data. Unlike the single decision tree, the RFC is a collection of decision-tree classifiers that generate a collection of decorrelated trees (random forest) based on multiple simulations of the actual training sample (Bhattacharyya et al., 2011; Breiman, 2001; Lokanan and Sharma, 2022; Papík and Papíková, 2022). The benefits of RFC over a single decision tree are increased stability, efficiency for large and small datasets, increased accuracy, robustness to noise, reduction of overfitting, adaptivity in handling multiple data attributes, and computational speed that is faster than other ensemble methods (Bhattacharyya et al., 2011; Breiman, 2001; Papík and Papíková, 2022; Wang and Song, 2011). Entropy is used to split the trees for the random forest model. Mathematically, entropy is represented in Equation 4:


(4) $$H\left(y\right)=-\text{plog}2\left(p\right)-\left(1-p\right)\log2\left(1-p\right)$$

where,

P is the probability of the +ve class,

(1-p) = The probability of the −ve class,

Hence, we are able to calculate H(y) for the dependent variable, financial distress.

#### 4.1.3 Support vector machines

SVM is a powerful tool for ML models and has been successfully used in a variety of tasks such as classification, regression, and outlier detection (Ghosh et al., 2019; Rtayli and Enneya, 2020). The SVM algorithm works by finding a hyperplane that best separates the data into classes. To do this, the algorithm first computes a set of support vectors, which are points in the data that are closest to the hyperplane (Botchey et al., 2020; Rtayli and Enneya, 2020). The distance between the support vectors and the hyperplane is called the margin (Rtayli and Enneya, 2020). The SVM algorithm then maximizes the margin to find the optimal decision boundary. When the data are linearly separable from the hyperplanes, then the SVM is represented by the linear formula in Equation 5:

Where


(5) $$B_{0}+B_{1}X_{1}+\ldots +B_{n}X_{n}=0$$

*B = (B_1_, … B_n_)*, and

*X = (X1, … Xn)* are *n*-dimensional vectors.

In the event that the data is linearly inseparable, then kernel tricks such as polynomial, Gaussian radial basis, sigmoid, and hyperbolic tangent are applied to map the data into a higher-dimensional space (Botchey et al., 2020). SVM has proven to be a robust algorithm for fraud detection tasks (Botchey et al., 2020; Rtayli and Enneya, 2020). Researchers have used SVM to predict data patterns that may indicate fraud and aberrant patterns in financial transactions (Botchey et al., 2020). By studying historical transaction data, SVM can learn to recognize “typical” transaction characteristics from abnormal transactions (Li et al., 2021; Rtayli and Enneya, 2020).

#### 4.1.4 ANN

**Figure 2:**
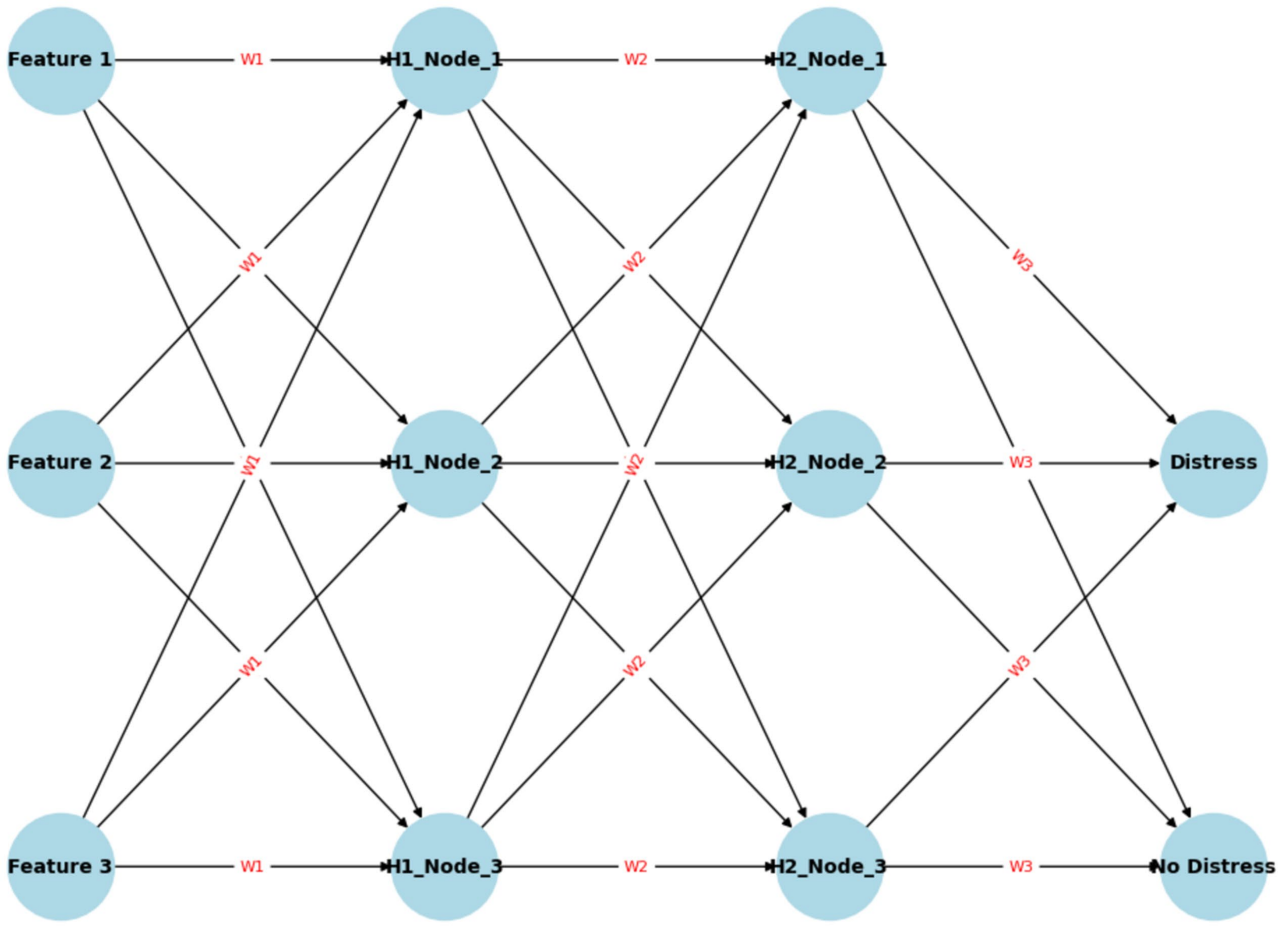
Neural network model.

ANN has been previously used in fraud prediction and financial distress research with very good performance (Bao et al., 2020; Lokanan, 2022; Mselmi et al., 2017). Owing to the presence of multiple neurons, ANN is a reliable algorithm because it can analyze large datasets, efficiently handle and process nonlinear data, and easily solve complicated tasks (Johnson and Khoshgoftaar, 2019; Lokanan, 2022). Figure 2 presents an illustration of a feedforward ANN. The neural network takes in three input features, processes them through two hidden layers, and produces a binary prediction (either “Distress” or “No Distress”) based on the activations of the neurons in the output layer. The feedforward method highlights factors influencing the output during every step in a neural network, thus allowing for improved predictive accuracy.

The network consists of an input layer representing features such as financial metrics (e.g., revenue, debt, profit margins), two hidden layers that transform inputs using weighted sums and biases to capture complex patterns, and an output layer with nodes indicating “Distress” and “No Distress.” The ANN operates as a feedforward neural network, where data flows from the input through the hidden layers to the output, with each connection between neurons having weights (W) and biases (b) to adjust during training. The role of each layer, the parameters involved, and the process of classification are explained to demonstrate how the ANN learns to identify distress through adjusting weights and biases. The mathematical representation of the feedforward ANN is shown in Equation 6:


(6) $$\sum i=1\;\left(W_{i}.X_{i}\right)+b=y$$

where,

∑_*i* = 1_ represents the summation of the weighted inputs from all connected neural networks.

*W*_*i*_ are the weights assigned to the connections between the input features (predictors) *X*_*i*_ and the neural network.

*b* is the bias or error term.

∑(*x*) is the function that computes the weighted sum of inputs and bias to predict the output *y*. This equation represents the basic operation of a neural network, where inputs are multiplied by their respective weights, summed up, and then passed through an activation function to produce the output.

#### 4.1.5 CART with bootstrapped samples

Bootstrapping is a specific resampling technique where you repeatedly draw random samples, with replacement, from your original dataset to create new samples of the same size as the original. These bootstrapped samples will be analyzed using CART to assess the robustness and stability of the model when applied to different variations of the data. The bootstrapped samples consist of various ratios ranging from 1:1 to 10:1 and represent different ratios of non-distress firms to financial distress firms in a dataset. Each sample is characterized by the ratio, the number of non-distress firms, and the number of financial distress firms. For instance, the first sample has a 1:1 ratio, meaning there are an equal number of non-distressed and financially distressed firms. Subsequent samples exhibit varying levels of class imbalance, with the ratio increasing from 2:1 to 10:1, indicating a decreasing proportion of financial distress firms relative to non-distressed firms. These bootstrapped samples allow for the exploration of model performance under different class distributions of the data, aiding in the evaluation and optimization of predictive models for financial distress. Furthermore, bootstrapping helps estimate the variability of model performance and allows the model to generalize effectively across different data subsets.

### 4.2 Performance measures

**Figure 3:**
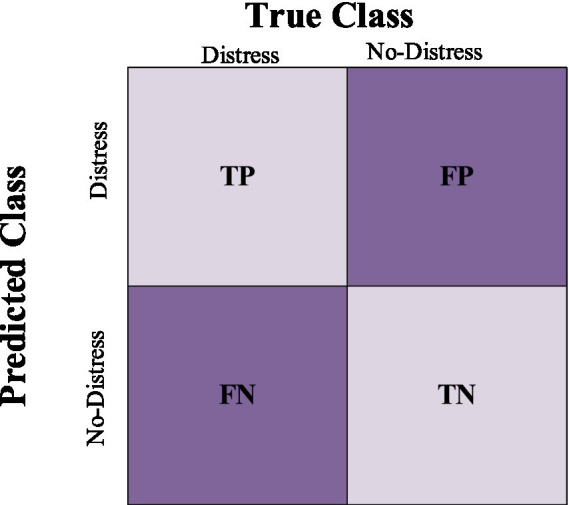
Confusion matrix.

The performance of a classifier can be visualized through a confusion matrix. As seen in Figure 3, the matrix is composed of four quadrants, each representing the predicted and actual values for one class. The quadrants are labeled true positive (TP), false positive (FP), true negative (TN), and false negative (FN). The true positive rate (TPR) is the proportion of cases correctly classified as positive, while the false positive rate (FPR) is the proportion of cases incorrectly classified as positive. The true negative rate (TNR) is the proportion of cases correctly classified as negative, while the false negative rate (FNR) is the proportion of cases incorrectly classified as negative. In general, a classifier with a high TPR and FPR will be more accurate than one with a lower TPR and higher FPR (Botchey et al., 2020; Lokanan and Liu, 2021; Lokanan, 2022).

The performance metrics used in this paper are shown in Table 3. Accuracy is the proportion of total observations that the model predicts correctly. Precision is a measure of accurate prediction. In the context of this paper, precision measures how often the classifier is correct when it predicts that a firm is experiencing financial distress. Precision attempts to answer the following question: *what proportion of the positive identifications was actually correct*? Recall is a measure of the completeness of the model. Recall measures how correct the model was in predicting actual financial distress and answers the following question: *what proportion of actual distressed firms were identified correctly*? The F-measure combines precision and recall into a single metric, while the receiver operating characteristics (ROC) curve visualizes the trade-off between sensitivity and specificity. Ultimately, the objective is to choose the model that strikes the best balance between accuracy and precision for deployment.

**Table 3:** Evaluation metrics.

Metrics	Correct formulae
Accuracy	TP + TN/(TP + TN + FP + FN)
Error rate	1 − Accuracy
Sensitivity (true positive rate, TPR)	TP/(TP + FN)
Specificity (true negative rate, TNR)	TN/(TN + FP)
Precision	TP/(TP + FP)
F1-score (F-measure)	2 × (Precision × Recall)/(Precision + Recall)
False positive rate (FPR)	FP/(FP + TN)
ROC area under the curve (AUC)	Plot TPR [TP/(TP + FN)] and FPR [FP/(TN + FP)] to a single score.

## 5 Experimental results

### 5.1 Summary statistics

Table 4 shows the summary results of the explanatory features and their effects on financial distress. While ratios from profitability, solvency, and operational performance show potential signs of financial distress, there are no specific signs in the liquidity and efficiency ratios that strongly indicate financial distress. In terms of profitability, the mean values for ROA, GPM, NPM, ROE, and NPTA are generally lower than zero, indicating potential financial distress. These ratios typically reflect a firm’s ability to generate profits in relation to its assets and equity. As for the solvency ratios, the mean values for DEQ, TLTA, and CLTA are relatively high, suggesting potential financial distress. Higher debt ratios and total liability ratios may indicate increased financial risks. The mean values of the operational performance ratios, namely EBITDAR, EBITDA, and EBIT, are relatively low compared to their respective maximum values. These findings indicate potential financial distress, as these ratios represent the firm’s operational profitability and ability to cover its operating expenses and debts.

**Table 4:** Summary results.

Index	ROA	GPM	NPM	ROE	NPTA	
Profitability						
Mean	−0.02	38.29	−15.56	−0.77	−0.02	
Std	0.28	35.5	177.52	18.75	0.28	
Min	−3.5	−376.67	−2156.49	−400.22	−3.5	
Max	0.85	127.05	818.22	32.99	0.85	
Liquidity						
Index	CR	QR	AccPT	CCL	WCTA	CTA
Mean	2.67	2.09	55.01	1.21	0.14	0.13
Std	4.06	3.03	114.29	2.44	0.22	0.16
Min	0.06	0.04	−271.39	0	−0.53	0
Max	49.67	22.9	1819.35	18.4	0.86	0.8
Efficiency						
Index	AT	InvT	AR	AP		
Mean	0.59	152.21	370684533.5	300977347.9		
Std	0.52	963.67	1,222,854,468	1,537,013,650		
Min	−0.43	0	0	0		
Max	3.81	18971.07	16,098,000,000	29,136,000,000		
Solvency						
Index	DEQ	TLTA	NPTL	CTL	CATA	CLTA
Mean	2.95	0.52	−0.02	0.62	0.34	0.2
Std	90.89	0.31	1.25	1.69	0.25	0.17
Min	−666.29	0.01	−6.47	0	0	0
Max	1835.89	3.7	16.4	13.98	0.99	1.25
Operational performance						
Index	EBITDAR	EBITDA	EBIT			
Mean	0.08	620425545.5	0.07			
Std	1.66	1,834,219,690	0.28			
Min	−20.37	−1,742,816,000	−3.26			
Max	7.62	20,331,000,000	0.87			

### 5.2 Control variables

Table 5 displays the results of the control variables. The results provide valuable insights into various factors that can potentially influence a company’s financial health and stability. Among these metrics, credit ratings and R_Score are key indicators of a firm’s overall financial health, with an average rating of 6.79 suggesting that many companies in the dataset are relatively stable. However, there is some variability, with scores ranging from 1 to 9, indicating the presence of both strong and distressed firms. When examining financial performance, Enterprise Value and Market Capitalization are critical. The substantial variation in these scores, with a mean Enterprise Value of approximately $6.1 billion and a mean Market Capitalization of around $4.3 billion, reflects differences in company sizes and valuations within the dataset. It is noteworthy that there are companies with negative Enterprise Value, which could indicate severe financial distress. Revenue and income growth are also vital for assessing a company’s performance. The average Revenue Growth of 0.87% suggests relatively modest revenue expansion, while the average Net Income Growth of 1.51% shows a similar trend in profitability. However, the wide standard deviations in these figures suggest significant variations among firms, with some experiencing negative growth, possibly indicating financial distress. Dividend growth is another critical factor. The mean value of 0.58 suggests that, on average, companies experienced positive dividend growth, which may be seen as a favorable financial indicator. However, the wide standard deviation and the presence of negative values (minimum of −1) indicate that there is significant variability in dividend growth rates, and some companies experienced a decline in dividends.

**Table 5:** Summary results control variables.

Index	Count	Mean	Std	Min	Max
Rating	463	6.79	2.51	1	9
R_Score	463	4.16	0.67	2	5
EV	463	6,085,702,971	19,364,571,360	−431,125,203	2.67566E+11
MarketCap	463	4,338,120,539	12,669,013,099	0	1.33198E+11
Rev_G	463	0.87	7.54	−4.81	156.6
NI_G	463	1.51	19.66	−270.21	174.81
D_G	463	0.58	3.92	−1	65.26
CG_Score	463	5.46	2.77	1	10
ARO	463	5.52	2.78	1	10
BoardS	463	5.42	2.77	1	10
ShareR	463	4.9	2.91	1	10
Comp	463	5.47	2.86	1	10

Corporate governance is fundamental to long-term stability (Farber, 2005; Nasir et al., 2019). The analysis of corporate governance measures (Corporate Governance Scores, Audit and Risk Oversight, Board Structure, Shareholder Relations, and Compensation Scores) indicates moderate to high scores on average but with significant variability among companies. These findings indicate that, on average, companies exhibit moderate to high scores in various aspects of corporate governance, such as the strengths of audit committees, board structure, executive compensation, and overall corporate governance practices (Basalat et al., 2023; Nour et al., 2024). However, the significant variability among companies in these measures suggests that financial distress is not solely determined by corporate governance scores. While stronger governance practices may exist on average, the presence of weaker governance in some companies implies that financial distress cannot be solely attributed to governance deficiencies (Basalat et al., 2023; Nasir et al., 2019; Nour et al., 2024). Other factors and circumstances likely play a substantial role in the occurrence of financial distress among firms.

Table 6 examines the financial ratios that indicate signs of financial distress in comparison to non-financially distressed firms. As expected, financially distressed firms show concerning signs. On average, these firms have a negative ROA of −0.05%, indicating inefficiency in generating profits from their assets. Although they have a positive GPM of 33.28%, the significantly negative NPM of −69.94% is alarming, pointing to significant losses. Interestingly, the ROE remains positive at 0.42%, indicating that these firms generate some returns for their equity holders. It is worth noting that some distressed firms maintain a negative DEQ ratio, implying a more equity-heavy capital structure. The negative EBITDAR of −0.42 suggests potential operational challenges. The results suggest that distressed firms generally struggle with profitability, incur substantial losses, and have a more conservative debt structure compared to their non-distressed counterparts. Non-distressed firms generally exhibit stronger profitability, more efficient operations, and a more stable financial position compared to their financially distressed counterparts. While these ratios show signs of potential financial distress, further statistical analysis is needed to confirm their predictive power.

**Table 6:** Summary results of distressed versus non-distressed firms.

	ROA	GPM	NPM	ROE	NPTA	DEQ	TLTA	CLTA	EBITDAR	EBITDA	EBIT
Financially distressed firms											
Mean	−0.05	33.28	−69.94	0.42	−0.05	−0.01	0.42	0.2	−0.42	5.92E+08	0.04
Std	0.32	−63.11	252.01	4.5	0.32	3.84	0.23	0.19	2.21	1.51E+09	0.34
Min	−1.3	−376.67	−1282.79	−3.3	−1.3	−27.71	0.05	0.02	−9.23	−3.81E+08	−0.92
Max	0.63	99.88	89.45	32.99	0.63	4.06	1.01	0.74	1.5	8.25E+09	0.87
Non-financially distressed firms											
Mean	−0.01	38.98	−8.08	−0.93	−0.01	3.36	0.54	0.2	0.15	6.24E+08	0.07
Std	0.28	29.84	163.69	19.93	0.28	96.93	0.32	0.17	1.57	1.88E+09	0.27
Min	−3.5	−193.02	−2156.49	−400.22	−3.5	−666.29	0.01	0	−20.37	−1.74E+09	−3.26
Max	0.85	127.05	818.22	27.88	0.85	1835.89	3.7	1.25	7.62	2.03E+10	0.87

We use the *t*-test to assess whether there is a statistically significant difference between distressed and non-distressed firms. Table 7 displays the results from the *t*-tests for distressed and non-distressed firms. The results from the *t*-test indicate that several financial ratios were found to be statistically significant in distinguishing distressed firms from non-distressed firms. These statistically significant ratios include the QR, NPM, CCL, TLTA, CTL, EBITDAR, and CRA. To put it into context, distressed firms exhibit a significantly higher quick ratio and higher cash-to-current liabilities, indicating potential liquidity challenges. They also tend to have significantly lower net profit margins, which imply operational difficulties. Additionally, distressed firms show a higher TLTA, suggesting a heavier debt burden. Distressed firms tend to have lower EBITDAR compared to non-distressed firms. On the other hand, non-distressed firms have a higher proportion of CTA and CTL, indicating a stronger cash position. These findings underscore the importance of considering these financial metrics when assessing the financial health and risk profiles of companies in both distressed and non-distressed scenarios.

**Table 7:** Results from *t*-tests.

Variable	Mean (distressed)	Mean (non-distressed)	T-statistic	*P*-value
ROA	−0.05	−0.01	−0.99	0.32
CR	3.59	2.55	1.81	0.07
QR	3.22	1.94	2.99	0.00
AccRT	92.81	80.56	0.56	0.58
AccPT	79.24	51.68	1.70	0.09
InvT	175.93	148.94	0.20	0.84
AT	0.58	0.59	−0.12	0.90
GPM	33.28	38.98	−1.13	0.26
NPM	−69.94	−8.08	−2.46	0.01
ROE	0.42	−0.93	0.50	0.61
CCL	2.00	1.11	2.59	0.01
NPTA	−0.05	−0.01	−0.99	0.32
NPTL	−0.12	−0.01	−0.63	0.53
DEQ	−0.01	3.36	−0.26	0.80
TLTA	0.42	0.54	−2.74	0.01
EBITDAR	−0.42	0.15	−2.40	0.02
EBIT	0.04	0.07	−0.78	0.44
WCTA	0.18	0.13	1.65	0.10
CTA	0.18	0.12	2.67	0.01
CTL	1.11	0.56	2.31	0.02
CATA	0.38	0.33	1.41	0.16
CLTA	0.20	0.20	−0.08	0.93
AR	231118971.91	389887657.39	−0.91	0.36
AP	160845429.63	320258398.04	−0.73	0.47
EBITDA	591939418.25	624345012.67	−0.12	0.90

### 5.3 Results from machine learning classifiers

The results from the predictive modeling experiments reveal insightful patterns. As shown in Table 8, all of the predictive models demonstrate high overall accuracy, with logistic regression achieving 91% accuracy, decision tree, random forest, and SVM all achieving 96% accuracy. However, it is worth noting that these models have varying performance in terms of precision, recall, and F1-score. Decision tree and random forest outperform logistic regression and SVM in terms of recall and F1-score, suggesting that they may have better capabilities to correctly identify distressed firms.

**Table 8:** Predictive modeling results.

Algorithm	Train accuracy	Test accuracy	Precision	Recall	F1-score
Linear, kernal, tree-based and ensemble modeling					
Logistic regression	0.91	0.91	1.00	0.23	0.38
Decision tree	0.98	0.96	1.00	0.92	0.96
Random forest	0.97	0.96	1.00	0.93	0.97
SVM	0.99	0.96	1.00	0.92	0.96
RFECV model					
Logistic regression	0.85	0.81	0.25	0.28	0.26
Decision tree	0.94	0.91	0.75	0.43	0.59
Random forest	0.94	0.91	0.75	0.43	0.59
SVM	0.92	0.91	0.76	0.44	0.60

The RFECV experiments indicate a slight decrease in overall accuracy across all algorithms. Logistic regression, decision tree, random forest, and SVM achieve accuracies of 81, 91, 91, and 91%, respectively. Notably, the precision, recall, and F1-score for logistic regression have decreased compared to the predictive model, indicating a potential loss of predictive power. Decision tree and random forest maintain relatively stable performance with strong precision and recall scores, suggesting their robustness in predicting financial distress.

These findings highlight the importance of balancing different evaluation metrics in predictive modeling for financial distress. Assessing the disparity between the training and testing scores, it appears that neither the predictive modeling nor the RFECV model displays indications of overfitting. While high accuracy is desirable, it is equally crucial to consider precision, recall, and the F1-score, as they provide insights into the model’s ability to correctly identify distressed firms and minimize false positives. The SVM, Decision Tree, and Random Forest models, with their balanced precision and recall scores, demonstrate their suitability for predicting financial distress, even in scenarios involving feature selection. Logistic regression, while initially accurate, may require careful feature selection to optimize its performance. Overall, these results emphasize the significance of algorithm selection and experimental engineering in developing effective predictive models for financial distress.

**Figure 4:**
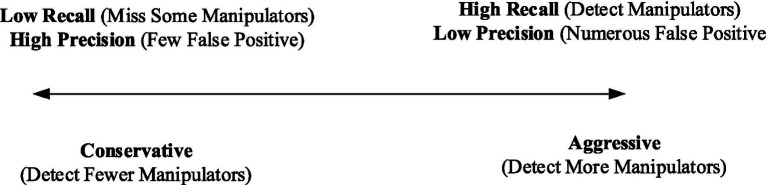
Approaches to detect distress in firms.

Detecting financial distress among firms boils down to professional judgment and domain knowledge. As shown in Figure 4, a more aggressive approach that flags a large number of firms as experiencing financial distress would have a high recall, as auditors would catch many instances of earnings manipulations that occur (Perols, 2011). However, this approach would also have low precision because it would falsely flag many healthy firms not involved in earnings manipulation as experiencing financial distress. In contrast, a highly conservative approach that only flags the most obvious cases of earnings management would likely have high precision (Perols and Lougee, 2011). However, it would overlook the more subtle instances of earnings manipulation and consequently have a lower recall. To strike the right balance between recall and precision, analysts need to carefully consider how they establish their systems and the criteria they use to flag potential cases of earnings manipulation or firms experiencing financial distress (Baryannis et al., 2019; Lokanan, 2022).

### 5.4 Results from ANN

As displayed in Table 9, the ANN model emerges as the most proficient algorithm for forecasting financial distress. The ANN model surpasses all other ML classifiers in performance, underscoring the potential for neural network models to enhance predictive accuracy in financial distress forecasting when contrasted with conventional classifiers. Notably, the recall score assumes particular significance in this context, as it effectively signifies the model’s capability to identify instances of financial distress, a vital aspect for company stakeholders aiming to preempt bankruptcy risks (Almaskati et al., 2021; Aviantara, 2021; Sun et al., 2021). The noticeable uptick in predictive accuracy offered by the ANN model, in comparison to its traditional ML classifiers, substantiates the rationale for corporations to persist in their investments in AI-based tools for financial distress prediction and detection.

**Table 9:** ANN performance.

ANN feedforward model	Precision	Recall	F1-score	Accuracy
Train scores	1.00	1.0	1.0	1.0
Test scores	0.98	0.96	0.97	0.98

### 5.5 CART with bootstrapping

Table 10 presents the outcomes derived from the CART model employing bootstrapped samples. The results indicate varying performance levels across different sample ratios, with the 3:01 and 6:01 ratios consistently outperforming the other samples. In this context, the ratio “3:01” means there are three normal transactions for every one financial distress transaction, helping to balance the dataset during model training. These two ratios consistently achieve higher test accuracy, test F1-scores, and test AUC values. Several factors could contribute to their superior performance, including the potential presence of more pertinent features for distinguishing between distressed and non-distressed firms or the model’s capacity to glean insights from the limited distressed samples. Additionally, dataset-specific characteristics or distinct financial behaviors exhibited by firms in the 3:01 and 6:01 ratios may favor the CART model’s predictive abilities. The model appears to generalize well to unseen data and accurately identify instances of financial distress. As the class imbalance ratio decreases, transitioning from 3:01 to 6:01, the models maintain their proficiency in accurately classifying the majority class (normal transactions) during training. Nevertheless, there is a slight decline in overall performance, particularly concerning the classification of the minority class (financial distress transactions). This decline is expected, as classifying the minority class becomes more challenging in the presence of a higher-class imbalance.

**Table 10:** Bootstrapped samples with CART.

Samples	Ratio	Normal transactions	Financial distress transactions	Training accuracy	Training F1-score	Training AUC	Test accuracy	Test F1-score	Test AUC
0	1:1	173	173	1.00	1.00	1.00	0.84	0.34	0.63
1	2:1	173	86	1.00	1.00	1.00	0.89	0.43	0.66
2	3:1	173	57	1.00	0.99	0.99	0.92	0.57	0.71
3	4:1	173	43	0.99	0.96	0.97	0.91	0.50	0.67
4	5:1	173	34	0.97	0.89	0.90	0.91	0.50	0.67
5	6:1	173	28	0.98	0.90	0.91	0.92	0.57	0.71
6	7:1	173	24	0.98	0.91	0.92	0.90	0.33	0.60
7	8:1	173	21	0.98	0.89	0.90	0.89	0.24	0.57
8	9:1	173	19	0.98	0.91	0.92	0.90	0.33	0.60
9	10:1	173	17	0.98	0.90	0.91	0.90	0.33	0.60

### 5.6 Feature relevance

**Figure 5:**
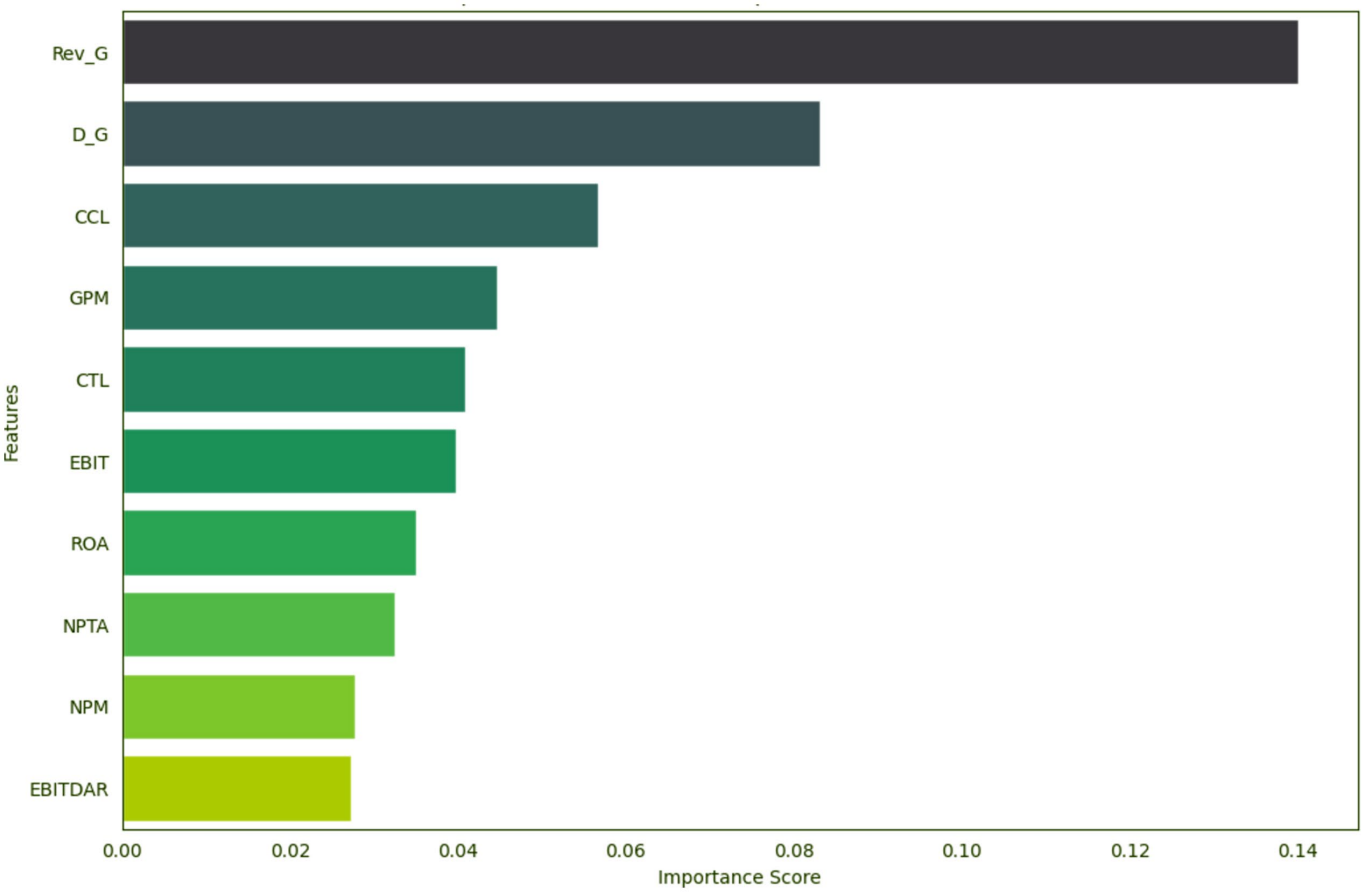
Top 10 feature relevance.

Figure 5 displays the coefficient analysis of the top 10 features for predicting financial distress, as determined by the random forest model. Revenue growth emerges as the most significant predictor, indicating that the rate of revenue growth strongly influences the likelihood of financial distress. Close behind is the dividend growth rate, which also plays a substantial role. A higher dividend growth rate can be a warning sign for potential financial instability, as it may suggest that the company is overcommitting to its shareholders at the expense of its financial stability. The random forest model’s ability to prioritize these features underscores their critical importance in the context of financial distress prediction.

Among the various financial ratios considered in the study, cash-to-current liabilities stands out as having a notable impact on the predictive accuracy of the models. This suggests that the amount of cash a company holds in relation to its current liabilities is a critical factor in assessing its financial stability. Additionally, the company’s profit margins stand out as an essential predictor. Lower GPM values may indicate that the company is struggling to maintain profitability, potentially leading to financial distress. The amount of cash available to pay off liabilities is also significant in predicting financial distress. A higher CTL ratio suggests that the company has sufficient cash to cover its liabilities, which can be a protective factor against distress. EBIT and EBITDAR are also prominent features. Their presence in the top 10 predictors underscores the importance of operational profitability for financial stability.

Table 11 provides insights into key features for predicting financial distress by analyzing the mean and standard deviation. Distressed firms tend to have higher mean values in revenue growth, but with significant variability. Their dividend growth rates are lower on average, with greater variations. On average, financially distressed companies tend to have a higher cash-to-current liabilities ratio compared to non-distressed companies. However, there is more variability in the CCL ratio among financially distressed companies, as indicated by the larger standard deviation. This finding may imply that having a relatively higher cash position in relation to current liabilities could be a financial characteristic somewhat associated with financial distress. Distressed firms have lower profitability, although with substantial variability. The CTL ratio suggests that distressed firms tend to have a higher cash reserve relative to total liabilities, but with greater variability. EBIT and EBITDAR ratios show minimal variations and minor differences in means. NPM stands out with significantly lower mean values and a wide standard deviation for distressed firms, highlighting their financial struggles and considerable profitability variation. These findings underscore the need for a nuanced financial distress prediction approach, considering central tendencies and variability.

**Table 11:** Top features mean and standard deviation.

Index	Feature	Non-financial distress	Distress
0	Rev_G	0.28 ± 1.17	5.14 ± 21.14
1	D_G	0.39 ± 2.50	1.92 ± 8.98
2	CCL	1.11 ± 2.19	2.00 ± 3.72
3	GPM	38.98 ± 29.84	33.28 ± 63.11
4	CTL	0.56 ± 1.58	1.11 ± 2.30
5	EBIT	0.07 ± 0.27	0.04 ± 0.34
6	ROA	−0.01 ± 0.28	−0.05 ± 0.32
7	NPTA	−0.01 ± 0.28	−0.05 ± 0.32
8	NPM	−8.08 ± 163.69	−69.94 ± 252.01
9	EBITDAR	0.15 ± 1.57	−0.42 ± 2.21

## 6 Discussion

In this study, we explored the potential of ML algorithms for predicting financial distress on the TSX using the Beneish M-score as a proxy for the target outcome. The TSX dataset includes a wide range of financial variables for listed companies, which, when evaluated using ML-based algorithms, can construct efficient predictive models. Our results reveal critical insights into the potential indicators of the financial health of companies listed on the TSX. Notably, revenue and dividend growth, cash-to-current liabilities, gross profit margins, and cash available to fund liabilities emerged as the top features for predicting financial distress. These findings align with prior research, such as Hajek and Henriques (2017), Mselmi et al. (2017), and Zhao et al. (2023), who also identified lower liquidity, profitability, and solvency ratios as key predictors of financial distress. However, our study contributes to the literature by providing a Canadian context, thereby broadening the geographical scope of financial distress prediction research, which has predominantly focused on Asian markets (Achakzai and Peng, 2023; Jiang and Jones, 2018).

Our results corroborate findings from global studies, such as those conducted in South Korea (Shaked and Altman, 2016; Bae, 2017), Taiwan (Huang and Yen, 2019), and the UAE (Sreedharan et al., 2020), which also emphasize the role of liquidity and profitability ratios in financial distress prediction. However, our study introduces new insights by highlighting the significance of revenue and dividend growth in the Canadian market, which may be reflective of unique economic conditions or corporate governance practices prevalent in Canada. These contrasts with other global studies underscore the need for context-specific models that can account for regional differences in financial behavior.

The integration of various ML algorithms, particularly the use of CART with bootstrapping and RFECV, underscores the importance of employing diverse methodologies to enhance the robustness of financial distress prediction models. Our findings suggest that while standalone ML models are effective, the combination of different techniques can lead to more accurate and interpretable outcomes. This study demonstrates that a multi-faceted approach to model development, which includes feature selection and dataset balancing, can significantly improve the prediction of financial distress.

One of the strengths of this study is the application of RFECV to systematically identify and select the most relevant features, enhancing both the interpretability and efficiency of the models. Additionally, the use of CART with bootstrapping provides a practical and transparent framework for predicting financial distress, particularly in the context of TSX-listed firms. However, the study also has limitations, including potential biases introduced by the selection of features and the possibility of overfitting due to the relatively small sample size. Furthermore, while our models performed well in a Canadian context, their generalizability to other markets may be limited, suggesting a need for further research in diverse geographical settings.

The findings from this study have important practical implications for auditors, regulators, and company management. By employing ML-based tools, these stakeholders can enhance the detection of financial distress and earnings manipulation, improving the accuracy and reliability of financial statements. The application of these predictive techniques to audit engagements can provide auditors with additional support, enabling them to react swiftly to signs of financial distress and protect the integrity of their engagements. Additionally, companies can use these models to proactively monitor their financial health, allowing them to take corrective actions before distress leads to more severe consequences.

## 7 Conclusion

Through the application of ML models to Canadian-listed businesses utilizing the Beneish M-score, this study has added to the expanding body of knowledge on financial distress prediction by closing the gap between conventional financial analysis and state-of-the-art data science approaches. This work addresses a significant knowledge gap by combining linear, kernel, tree-based, and ensemble algorithms, recursive feature elimination with cross-validation (RFECV), and bootstrapped classification and regression trees (CART) to understand how advanced algorithms can improve the detection of financial distress. An important addition to this research is the identification of pivotal indicators, including revenue growth, dividend growth, cash-to-current obligations, and gross profit margins, that greatly impact the probability of financial distress. Empirically, the results emphasize the efficacy of artificial neural networks (ANN) and ensemble models, such as random forests, in accurately forecasting distressed companies. A more proactive approach to monitoring business financial health is made possible by these sophisticated models, which provide auditors, regulators, and firm management with actionable data. The study’s usage of CART with bootstrapped samples offers an innovative method that improves the resilience and dependability of the model, hence assuring better generalization across various datasets.

Furthermore, the research underscores the distinctive circumstances of the Canadian market, underscoring that the increase in revenue and dividends are crucial measures of financial stability in this region. The findings of this study are profound for policymakers and regulators in Canada, as it indicates the need to develop region-specific prediction models that take into consideration the local economic and corporate governance circumstances. Through these sophisticated methodologies, Canadian authorities and auditors may enhance their ability to identify fraudulent activities, guaranteeing the highest adherence to financial reporting requirements. This equips policymakers with the necessary data to improve monitoring systems and decrease occurrences of earnings manipulation and financial misreporting. Future research should enhance the dataset by integrating macroeconomic statistics, market sentiment data, and industry-specific measures to construct more extensive prediction models. Moreover, using time-series data to analyze financial distress enables a more profound understanding of the temporal evolution of a company’s financial state. In addition, researchers should investigate the worldwide relevance of these results by doing comparable studies in other geographical areas and sectors to evaluate the resilience of the constructed models in other settings. Although this research furnishes useful insights, it is subject to various limitations. The dataset is restricted to measures of financial ratios and variables at the business level, which may not include the complexity of financially distressed situations. Furthermore, the research just examines one nation, restricting the results’ applicability to other markets. Moreover, the limited sample size and the restricted time period of the dataset (which coincides with the COVID-19 epidemic) can add biases, therefore impacting the transferability of the findings to other economic settings.

## Data Availability

The raw data supporting the conclusions of this article will be made available by the authors, without undue reservation.
