# Supplementary material for: Predicting financial distress in TSX-listed firms using machine learning algorithms
Source: Front Artif Intell. 2024 Nov 27;7:1466321. doi: 10.3389/frai.2024.1466321 (PMC11631907; doi:10.3389/frai.2024.1466321)
Supplement: Supplementary file 1 [file Table_1.docx]

**Appendix 1**

**Independent Variables**

| **Variables** | **Description** | **Papers** |
| --- | --- | --- |
|  | **Independent Variables** |  |
| **Profitability Ratios** | | |
| ROA | Return on Assets | Demetriades & Owusu-Agyei, 2022; Fang et al., 2017; Gupta & Mehta, 2020; Hajek & Henriques, 2017; Song et al., 2014; Sun et al., 2014 |
| ROE | Return on Equity | Albizri et al., 2019; Craja et al., 2020; Gupta & Mehta, 2020; Huang et al., 2017 |
| NPM | Net Profit Margin | Albizri et al., 2019; Craja et al., 2020; Fang et al., 2017; Gupta & Mehta, 2020; Hajek & Henriques, 2017; Huang et al., 2017; Song et al., 2014 |
| GPM | Net Profit Margin |  |
| NPTA | Net Profit to Fixed Assets | Albizri et al., 2019; Gupta & Mehta, 2020 |
| **Liquidity Ratios** | | |
| CR | Current Ratio | Albizri et al., 2019; Fang et al., 2017; Gupta & Mehta, 2020; Hajek & Henriques, 2017; Huang et al., 2017; Song et al., 2014 |
| QR | Quick Ratio | Albizri et al., 2019; Fang et al., 2017; Gupta & Mehta, 2020; Hajek & Henriques, 2017; Huang et al., 2017; Song et al., 2014 |
| AccRT | Accounts Receivable Turnover in Days | Fang et al., 2017; Gupta & Mehta, 2020; Hajek & Henriques, 2017; Huang et al., 2017; Song et al., 2014 |
| WCTA | Working Capital to Total Assets | Albizri et al., 2019 |
| CCL | Cash to Current Liabilities | Albizri et al., 2019; Gupta & Mehta, 2020 |
| CTA | Cash to Total Assets | Albizri et al., 2019; Gupta & Mehta, 2020 |
| **Efficiency Ratios** | | |
| AT | Asset Turnover | Albizri et al., 2019; Fang et al., 2017; Gupta & Mehta, 2020; Huang et al., 2017 |
| InvT | Inventory Turnover | Zainudin & Hashim, 2016; Dimitropoulos & Asteriou (2009) |
| AR | Accounts Receivable Turnover in Days | Shaked & Altman, 2016 |
| AP | Accouns Payable Turnover in Days | Habib et al., 2018 |
| **Solvency Ratios** | | |
| DEQ | Debt to Equity | Albizri et al., 2019; Craja et al., 2020; Gupta & Mehta, 2020; Huang et al., 2017 |
| TLTA | Liabilities to Assets | Albizri et al., 2019; Gupta & Mehta, 2020 |
| NPTL | Net Profit to Total Liabilities | Craja et al. 2020 |
| CTL | Cash to Total Liabilities | Albizri et al., 2019; Gupta & Mehta, 2020 |
| CATA | Current Assets to Total Assets | Albizri et al., 2019; Fang et al., 2017; Gupta & Mehta, 2020 |
| CLTA | Current Liabilities to Total Assets | Albizri et al., 2019; Gupta & Mehta, 2020 |
| **Operating Performance** | | |
| EBIT | Earnings Before Interest and Taxes | Albizri et al., 2019 |
| EBITDAR | Earnings Before Interest, Taxes, Depreciation, Amortization, and Rent | Li, 2016 |
| EBITDA | Earnings Before Interest, Taxes, Depreciation, and Amortization | Campa & Camacho-Miñano, 2015 |
